# Solutions of the Multivariate Inverse Frobenius–Perron Problem

**DOI:** 10.3390/e23070838

**Published:** 2021-06-30

**Authors:** Colin Fox, Li-Jen Hsiao, Jeong-Eun (Kate) Lee

**Affiliations:** 1 Department of Physics, University of Otago, Dunedin 9016, New Zealand; 2 System Manufacturing Center, National Chung-Shan Institute of Science & Technology, New Taipei City 237209, Taiwan; hslijen@gmail.com; 3 Department of Statistics, The University of Auckland, Auckland 1010, New Zealand; kate.lee@auckland.ac.nz

**Keywords:** inverse Frobenius–Perron problem, Rosenblatt transformation, uniform map, multivariate probability distribution, transfer operator, ergodic map

## Abstract

We address the inverse Frobenius–Perron problem: given a prescribed target distribution ρ, find a deterministic map *M* such that iterations of *M* tend to ρ in distribution. We show that all solutions may be written in terms of a factorization that combines the forward and inverse Rosenblatt transformations with a uniform map; that is, a map under which the uniform distribution on the *d*-dimensional hypercube is invariant. Indeed, every solution is equivalent to the choice of a uniform map. We motivate this factorization via one-dimensional examples, and then use the factorization to present solutions in one and two dimensions induced by a range of uniform maps.

## 1. Introduction

A basic question in the theory of discrete dynamical systems, and in statistical mechanics, is whether a chaotic iterated function M:X→X that maps a space $X\subseteq R^{d}$ back onto *X* has an equilibrium distribution with probability density function (PDF) ρ(x). The PDF is with respect to some underlying measure, typically Lebesgue. Throughout this paper, we use the same symbol for the distribution and associated PDF, with meaning taken from context.

A necessary condition is that the distribution ρ is *invariant* under *M*; i.e., if x∼ρ (*x* is distributed as ρ), then so is M(x), and further that the orbit of almost all points x∈X defined as $O^{+}\left(x\right)=\left\{x,M\left(x\right),M^{2}\left(x\right),M^{3}\left(x\right),\ldots \right\}$ tends in distribution to ρ. Then, under mild conditions, the map is ergodic for ρ; that is, expectations with respect to ρ may be replaced by averages over the orbit [1,2].

For example, it is well known [1,2,3,4] that the logistic map $M_{\log}\left(x\right)=4x\left(1-x\right)$, for x∈[0,1], is chaotic with the equilibrium distribution having PDF

$$\rho_{\log}\left(x\right)=\frac{1}{\pi \sqrt{x(1-x)}},$$

implying that $M_{\log}$ is ergodic for $\rho_{\log}$.

Our motivating interest is the use of this ergodic property to implement sample-based inference for Bayesian analysis or machine learning. In those settings, the target distribution ρ is defined by the application. Generating a sequence $\left\{x_{0},x_{1},x_{2},x_{3},\ldots \right\}$ that is ergodic for ρ is useful because expectations of any quantity of interest can then be computed as averages over the sequence; i.e.,

(1) $$\lim_{N\to \infty }\frac{1}{N}\sum_{i=0}^{N-1}g\left(x_{i}\right)=\int_{X}g\left(x\right)\rho \left(x\right)\;dx.$$

In statistics, such ergodic sequences are commonly generated using *stochastic* iterations that simulate a Markov chain targeting ρ [5]; here, we explore *deterministic* iterations that generate an orbit that is ergodic for ρ.

The equilibrium distribution for a given iterated function, if it exists, can be approximated by computing the orbit of the map for some starting point and then performing kernel density estimation, or theoretically by seeking the stationary distribution of the Frobenius–Perron (FP) operator that is the transfer [6], aka push-forward, operator induced by a deterministic map [1,2]; we present the FP equation in Section 2.

The inverse problem that we consider here—determining a map that gives a prescribed equilibrium distribution—is the inverse Frobenius–Perron problem (IFPP) and has been studied extensively [7,8,9,10,11,12,13,14]. Summaries of previous approaches to the IFPP are presented in [12,15], which characterize approaches as based on conjugate functions (see [7] for details) or the matrix method (see [15] for details), and [16], which also lists the differential equation approach. Existing work almost solely considers the IFPP in one variable, d=1, with the exception being the development of a two-dimensional solution in [12] that is also presented in [15].

The matrix method, first suggested by Ulam [17], solves the IFPP for a piecewise-constant approximation to the target density using a transition matrix representation of the approximated FP operator. Convergence of the discrete approximation is related to Ulam’s conjecture and has been proved for the multidimensional problem; see [16] and references therein. While the matrix method allows the construction of solutions, at least in the limit, existing methods only offer a limited class of very non-smooth solutions, which are not clearly useful for characterizing all solutions, as we do here. We do not further consider the matrix methods.

The development in this paper starts with the differential equation approach in which the IFPP for restricted forms of distributions and maps is written as a differential equation that may be solved. We re-derive some existing solutions to the IFPP in this way in Section 3. The main contribution of this paper is to show that the form of these solutions may be generalized to give the general solution of the IFPP for any probability distribution in any dimension *d*, as presented in the factorization theorem of Section 4. This novel factorization represents solutions of the IFPP in terms of the Rosenblatt transformation [18] and a *uniform map*; that is, a map on ${\left[0,1\right]}^{d}$ that leaves the uniform distribution invariant. In particular, we show that the conjugating functions in [7] are exactly the inverse Rosenblatt transformations. For a given Rosenblatt transformation, there is a one-to-one correspondence between the solution of the IFPP and the choice of a uniform map.

This reformulation of the IFPP in terms of two well-studied constructs leads to practical analytic and numerical solutions by exploiting existing, well-developed methods for Rosenblatt transformations and deterministic iterations that target the uniform distribution. The factorization also allows us to establish the equivalence of solutions of the IFPP and other methods that employ a deterministic map within the generation of ergodic sequences. This standardizes and simplifies existing solution methods by showing that they are special cases of constructing the Rosenblatt transformation (or its inverse) and the selection of a uniform map.

This paper starts with definitions of the IFPP and the Lyuapunov exponent in Section 2. Solutions of the IFPP in one dimension, d=1, are developed in Section 3. These solutions for d=1 motivate the factorization theorem in Section 4, which presents a general solution to the IFPP for probability distributions with domains in $R^{d}$ for any *d*. Section 5 presents further examples of univariate, d=1, solutions to the IFPP based on the factorization theorem in Section 4. Two two-dimensional numerical examples are presented in Section 6.3 to demonstrate that the theoretical constructs may be implemented in practice. A summary and discussion of results is presented in Section 7, including a discussion of some existing computational methods that can be viewed as implicitly implementing the factorization solution of the IFPP presented here.

## 2. Inverse Frobenius–Perron Problem and Lyapunov Exponent

In this section, we define the forward and inverse Frobenius–Perron problems, and also the Lyuapunov exponent that measures chaotic behavior.

### 2.1. Frobenius–Perron Operator

A deterministic map $x_{n+1}=M\left(x_{n}\right)$ defines a map on probability distributions over state, called the transfer operator [6]. Consider the case where the initial state $x_{0}∼\rho_{0}\left(\cdot \right)$ ($x_{0}$ is distributed as $\rho_{0}$) for some distribution $\rho_{0}$ and let $\rho_{n}$ denote the *n*-step distribution; i.e., the distribution over $x_{n}=M^{n}\left(x_{0}\right)$ at iteration *n*. The transfer operator that maps $\rho_{n}\mapsto \rho_{n+1}$ induced by *M* is given by the Frobenius–Perron operator associated with M:x↦y [1,2,19]

(2) $$\rho_{n+1}\left(y\right)=\sum_{x\in M^{-1}\left(y\right)}\frac{\rho_{n}\left(x\right)}{\left|J(x)\right|}$$

where |J(x)| denotes the Jacobian determinant of *M* at *x*, and the sum is over inverse images of *y*. We have used the language of differential maps, as all the maps that we display in this paper are differentiable almost everywhere [20]. More generally, ${\left|J\left(x\right)\right|}^{-1}$ denotes the density of $\rho_{n}M^{-1}$ with respect to $\rho_{n+1}$; see, e.g., [2] (Remark 3.2.4.).

The equilibrium distribution ρ of *M* satisfies

(3) $$\rho \left(y\right)=\sum_{x\in M^{-1}\left(y\right)}\frac{\rho (x)}{\left|J(x)\right|}$$

and we say that ρ is invariant under *M*.

### 2.2. Inverse Frobenius–Perron Problem

The inverse problem that we address is finding an iterative map *M* that has a given distribution ρ as its equilibrium distribution. We do this by exploring the inverse Frobenius–Perron problem (IFPP) of finding an *M* that satisfies (3) to ensure that ρ is an invariant distribution of *M*. Establishing chaotic and thus ergodic behavior is a separate calculation.

We assume throughout this work that ρ is absolutely continuous with respect to the underlying measure, meaning that a probability density function ρ(x) exists and furthermore that ρ(x)>0, ∀x∈X.

### 2.3. Lyapunov Exponent

The Lyapunov exponent *h* of an iterative map gives the average exponential rate of divergence of trajectories. We define the (maximal) Lyapunov exponent *h* as [1,2]

(4) $$h=\lim_{N\to \infty }\frac{1}{N}\log\left|\frac{dx_{N}}{dx_{0}}\right|=\lim_{N\to \infty }\frac{1}{N}\sum_{n=0}^{N-1}\log\left|J\left(x_{n}\right)\right|$$

that features the starting value $x_{0}$. For ergodic maps, the dependency on $x_{0}$ is lost as N→∞, and the Lyapunov exponent may be written

(5) $$h=\int_{X}\log\left|J\left(x\right)\right|\rho \left(x\right)\;dx$$

where ρ(x) is the invariant density. A positive Lyapunov exponent *h* indicates that the map is chaotic.

The theoretical value for the Lyapunov exponent may be obtained using (5), while (4) provides an empirical value obtained by iterating the map *M*. For example, the Lyapunov exponent of the logistic map evaluated by (5) is $h_{\log}=\log2\approx 0.693147$, while (4) evaluated over an orbit with 10,000 iterations gives $h_{\log}\approx 0.693140$.

For chaotic maps, any uncertainty in the initial value means that an orbit cannot be precisely predicted, since initial states with any separation become arbitrarily far apart, within *X*, as iterations increase. It is therefore useful to characterize the orbit statistically, in terms of the equilibrium distribution over states in the orbit.

It is interesting to note that theoretical chaotic and ergodic behavior does not necessarily occur when iterations of a map are implemented on a finite-precision computer. For example, when the logistic map, as shown above, on [0,1] is iterated on a binary computer, the multiplication by 4 corresponds to a shift left by 2 bits, and all subsequent operations maintain lowest order bits that are 0. Repeated iterations eventually produce the number zero, no matter the starting value. While it is simple to correct this non-ideal behavior, as was done to give the numerical Lyapunov exponent, as shown above, it is important to note that computer implementation can have very different dynamics to the mathematical model.

## 3. Solution of the IFPP in 1-Dimension

In this section, we develop solutions of the IFPP in one dimension, d=1. Without loss of generality, we consider distributions on the unit interval X=[0,1] as the domain of any univariate distribution may be transformed by a change of variable to X=[0,1], including when the domain is the whole real line (−∞,∞).

For distributions over a scalar random variable, the FP equation for the invariant density (3) simplifies to

(6) $$\rho \left(y\right)=\sum_{x\in M^{-1}\left(y\right)}\frac{\rho \left(x\right)}{|M^{\prime }\left(x\right)|}.$$

### 3.1. The Simplest Solution

We first note, almost trivially, that the identity map M=I, where I(x)=x, has ρ as an invariant distribution and thus solves the IFPP for any ρ. Somewhat less trivial is the derivation of this simplest solution by assuming that *M* is monotonic increasing and M(0)=0, meaning that there is only one inverse image in (6). Writing $|M^{\prime }\left(x\right)|=dM/dx$ gives the differential equation with separated variables

(7) ρ(M)dM=ρ(x)dx

that has solution

F(M)=F(x)

where $F\left(x\right)=\int_{0}^{x}\rho \left(x^{\prime }\right)\;dx^{\prime }$ is the cumulative distribution function (CDF) for ρ. If *F* is invertible, denote the inverse by $F^{-1}$, called the the inverse distribution function (IDF); otherwise, let $F^{-1}$ denote the generalized inverse distribution function, $F^{-1}\left(p\right)=\inf\left\{x\in X:F\left(x\right)\geq p\right\}$. Then, $M=F^{-1}\left(F\left(x\right)\right)=x$, or M(x)=x, almost everywhere. Thus, the identity map is the *unique* monotonic increasing map that has ρ as its invariant distribution. Clearly, the identity map is not ergodic for ρ.

We may generalize this solution by setting M(0)=k, for some k∈[0,1), and also only requiring *M* to be piecewise continuous. Allowing one discontinuity in *M*, we write the integral of the separated differential Equation (7) as

**Figure 1:**
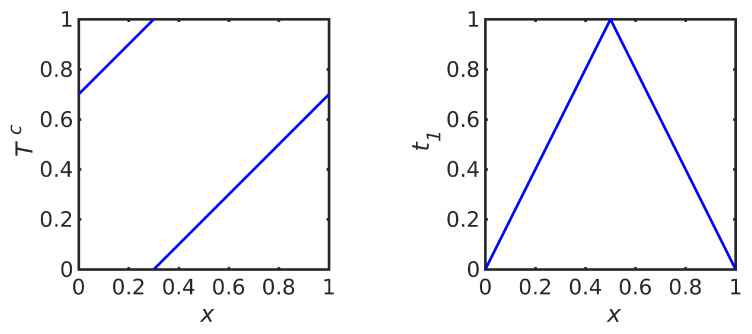
Translation operator $T^{c}$ for c=0.3 (**left**) and triangle map $t_{1}$ (**right**).

F(M)=F(x)+kmod1

giving the solution to the IFPP

(8) $$M\left(x\right)=\left(F^{-1}\circ T^{c}\circ F\right)\left(x\right)$$

where $c=F^{-1}\left(k\right)$. Here, $T^{c}$ denotes the operator that translates by *c* with a wrap-around on [0,1) (thus, $T^{c}$ is the translation operator on the unit circle $S^{1}$)

(9) $$T^{c}\left(y\right)=y+c-\left\lfloor y+c\right\rfloor ,$$

where ⌊⌋ denotes the floor function; see Figure 1 (left). The identity map is recovered when k=c=0.

It is easily seen that the Lyapunov exponent of the map in (8) is h=0, so the map is not chaotic. However, interestingly, this map can generate a sequence of states that produce a numerical integration rule with respect to ρ, since appropriate choices of *c* and the number of iterations *N* can produce a rectangle rule quadrature or a quasi Monte Carlo lattice rule; see Section 4.3.

### 3.2. Exploiting Symmetry in ρ(x)

When the PDF ρ has reflexive symmetry about 1/2—i.e., ρ(x)=ρ(1−x)—we can simplify the FP Equation (6) by assuming that the map *M* has the same symmetry. Specifically, we write the triangle map (see Figure 1 (right))

(10) $$t_{1}\left(x\right)=1-2\left|x-1/2\right|$$

that has reflexive symmetry about 1/2, and write

(11) $$M\left(x\right)=m(t_{1}\left(x\right))$$

where m(x):[0,1]→[0,1] is a monotonic increasing map with m(0)=0 (and, as is shown below, m(1)=1). Thus, the FP equation simplifies to

(12) $$\rho \left(y\right)=2\frac{\rho \left(x\right)}{|M^{\prime }\left(x\right)|},\;x\in M^{-1}\left(y\right),$$

which we can write as the separated equations

$$\begin{array}{cc} \rho (M)\;dM=2\rho (x)dx,\; & x<1/2,\\ \rho (M)\;dM=-2\rho (x)dx,\; & x>1/2, \end{array}$$

which have the continuous solution

$$F\left(M\right)=t_{1}\left(F\left(x\right)\right)$$

giving the continuous solution to the IFPP

(13) $$M\left(x\right)=\left(F^{-1}\circ t_{1}\circ F\right)\left(x\right).$$

One can solve for $m\left(x\right)=F^{-1}\left(2F\left(x/2\right)\right)$, though we do not further consider the function *m*.

The approach we have used here simplifies the approach in [9], while “doubly symmetric” maps of the form (11) were considered in [21] and again in [10,11].

### 3.3. Symmetric Triangular Distribution

**Figure 2:**
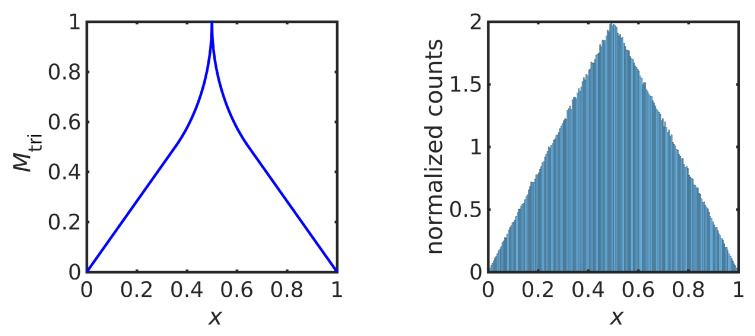
Iterative map $M_{\mathrm{tri}}$ in (15) (**left**) and a histogram of $1\times 10^{6}$ iterates of the map $M_{\mathrm{tri}}$ (**right**).

To give a concrete example of the solution in (13), we consider the symmetric triangular distribution on [0,1] with PDF

(14) $$\rho_{\mathrm{tri}}\left(x\right)=2-4\left|x-\frac{1}{2}\right|$$

that has reflexive symmetry about $x=\frac{1}{2}$. The CDF is

$$F_{\mathrm{tri}}\left(x\right)=\left\{\begin{array}{cc} 2x^{2} & 0\leq x\leq \frac{1}{2}\\ \frac{1}{2}+4x-2x^{2} & \frac{1}{2}\leq x\leq 1 \end{array}\right.$$

giving the unimodal map, after substituting into (13),

(15) $$M_{\mathrm{tri}}\left(x\right)=\left\{\begin{array}{cc} \sqrt{2}x & 0\leq x\leq \frac{1}{\sqrt{8}}\\ 1-\sqrt{\frac{1}{2}-2x^{2}} & \frac{1}{\sqrt{8}}\leq x\leq \frac{1}{2}\\ 1-\sqrt{\frac{1}{2}-2{\left(1-x\right)}^{2}} & \frac{1}{2}\leq x\leq 1-\frac{1}{\sqrt{8}}\\ \sqrt{2}\left(1-x\right) & 1-\frac{1}{\sqrt{8}}\leq x\leq 1 \end{array}\right.$$

shown in Figure 2 (left). The same map was derived in [7]. Figure 2 (right) shows a normalized histogram of $10^{6}$ iterations of $M_{\mathrm{tri}}$ starting at x=0.3, confirming that the orbit of $M_{\mathrm{tri}}$ converges to the desired triangular distribution. The numerical implementation avoids finite-precision effects, as discussed later.

The theoretical Lyapunov exponent for $M_{\mathrm{tri}}$ is $h_{\mathrm{tri}}=\log2\approx 0.693147$, while (4) evaluated over an orbit with $10^{6}$ iterations gives $h_{\mathrm{tri}}\approx 0.693148$.

## 4. Solutions of the IFPP for General Multi-Variate Target Distributions

The solutions to the one-dimensional IFPP with a special structure in Equations (8) and (13) are actually examples of a general solution to the IFPP for multi-variate probability distributions with no special structure. We state that connection via a theorem that establishes a factorization of all possible solutions to the IFPP and that also provides a practical means of solving the IFPP.

We first introduce the forward and inverse Rosenblatt transformations; that is, the multi-variate generalization of the CDF and IDF for univariate distributions.

### 4.1. Forward and Inverse Rosenblatt Transformations

A simple transformation of an absolutely continuous *d*-variate distribution into the uniform distribution on the *d*-dimensional hypercube was introduced by Rosenblatt [18], as follows. The joint PDF can be written as a product of conditional densities,

$$\rho \left(x_{1},\ldots ,x_{d}\right)=\rho_{1}\left(x_{1}\right)\rho_{2}\left(x_{2}|x_{1}\right)\cdots \rho_{d}\left(x_{d}|x_{1}\ldots ,x_{d-1}\right),$$

where $\rho_{k}\left(x_{k}|x_{1}\ldots ,x_{k-1}\right)$ is a conditional density given by

(16) $$\rho_{k}\left(x_{k}|x_{1}\ldots ,x_{k-1}\right)=\frac{p_{k}\left(x_{1},\ldots ,x_{k}\right)}{p_{k-1}\left(x_{1}\ldots ,x_{k-1}\right)},$$

in terms of the marginal densities,

(17) $$p_{k}=\int \rho \left(x_{1},\ldots ,x_{d}\right)\;dx_{k+1}\cdots dx_{d},$$

where k=1,…,d.

Let $z=\left(z_{1},\ldots ,z_{d}\right)=R\left(x_{1},\ldots ,x_{d}\right)$ where *R* is the Rosenblatt transformation [18] from the state-space $X\subseteq R^{d}$ of ρ to the *d*-dimensional unit cube ${\left[0,1\right]}^{d}$, defined in terms of the (cumulative) distribution function *F* by

$$\begin{array}{cc} z_{1} & =F_{1}\left(x_{1}\right)=\int_{-\infty }^{x_{1}}\rho_{1}\left(x_{1}^{\prime }\right)\;dx_{1}^{\prime },\\ z_{2} & =F_{2}\left(x_{2}|x_{1}\right)=\int_{-\infty }^{x_{2}}\rho_{2}\left(x_{2}^{\prime }|x_{1}\right)\;dx_{2}^{\prime },\\ \; & \vdots \\ z_{d} & =F_{d}\left(x_{2}|x_{1},\ldots ,x_{d-1}\right)=\int_{-\infty }^{x_{d}}\rho_{d}\left(x_{d}^{\prime }|x_{1},\ldots ,x_{d-1}\right)\;dx_{d}^{\prime }. \end{array}$$

As noted in [18], there are d! transformations of this type, corresponding to the d! ways of ordering the coordinates. Further multiplicity is introduced by considering coordinate transformations, such as rotations.

Notice that in one dimension the Rosenblatt transformation R(x) is simply the CDF F(x).

It follows that if x∼ρ, then $z=R\left(x\right)∼\mathrm{Unif}({\left[0,1\right]}^{d})$; i.e., *z* is uniformly distributed on the *d*-dimensional unit cube [18]. When ρ(x)>0, ∀x∈X, the distribution functions are strictly monotonic increasing and the inverse of the Rosenblatt transformation $R^{-1}$ is well defined; otherwise, let $R^{-1}$ denote the generalized inverse as in Section 3.1. Then, if $z∼\mathrm{Unif}({\left[0,1\right]}^{d})$, it follows that $x=R^{-1}\left(z\right)∼\rho$; i.e., *x* is distributed as the desired target distribution ρ [22]. This is the basis of the *conditional distribution method* for generating multi-variate random variables, which generalizes the inverse cumulative transformation method for univariate distributions [22,23,24,25]. These results may also be established by substituting *R* or $R^{-1}$ into the the FP Equation (2), noting that there is a single inverse image and that the Jacobian determinant of *R* equals the target PDF ρ(x).

In the remainder of this paper, we refer to *any* map *R* satisfying $x∼\rho \Rightarrow R\left(x\right)∼\mathrm{Unif}({\left[0,1\right]}^{d})$ as a Rosenblatt transformation, with the (generalized) inverse as defined above.

### 4.2. Factorization Theorem

The following theorem characterizes solutions to the IFPP.

**Theorem 1.**  *Given a probability distribution ρ in d dimensions, a map M(x) is a solution of the IFPP; that is, M(x) satisfies the FP Equation (3) if and only if*

(18) $$M\left(x\right)=\left(R^{-1}\circ U\circ R\right)\left(x\right),$$

*where R is a Rosenblatt transformation and U is a “uniform map” on the unit d-dimensional hypercube; i.e., a map that has $\mathrm{Unif}({\left[0,1\right]}^{d})$ as invariant distribution.*

**Proof.**  We show that ρ is an invariant distribution of *M* if and only if *M* has the form (18). (⇒) Assume *M* has the form (18). If x∼ρ, then $R\left(x\right)∼\mathrm{Unif}({\left[0,1\right]}^{d})$; thus, $U\left(R\left(x\right)\right)∼\mathrm{Unif}\left({\left[0,1\right]}^{d}\right)$, as $\mathrm{Unif}({\left[0,1\right]}^{d})$ in invariant under *U*, and thus $M\left(x\right)=R^{-1}\left(U\left(R\left(x\right)\right)\right)∼\rho$, as desired. (⇐) If ρ is invariant under *M*, then $U=R\circ M\circ R^{-1}$ is a uniform map, since if $z∼\mathrm{Unif}({\left[0,1\right]}^{d})$, then $R^{-1}\left(z\right)∼\rho$, $M(R^{-1}\left(z\right))∼\rho$, and $R\left(M\left(R^{-1}\left(z\right)\right)\right)∼\mathrm{Unif}\left({\left[0,1\right]}^{d}\right)$. Inserting this *U* into (18) gives the desired factorization. □

The first part of the proof shows that any uniform map *U* induces a solution to the IFPP, though the particular solution depends on the particular Rosenblatt transformation. The second part of the proof shows that different solutions to the IFPP effectively differ only by the choice of the uniform map *U*, once the Rosenblatt transformation is determined; that is, a coordinate system is chosen with an ordering of those coordinates.

Grossmann and Thomae [7] referred to dynamical systems *M* and *U* related by a formula of the form (18) as “related by conjugation”, or simply “conjugate”, and the map $R^{-1}$ in (18) is a “conjugating function”. Thus, in the language of [7], Theorem 1 shows that the IFPP for any distribution ρ has a solution (actually, it shows that there are infinitely many solutions), every solution map is conjugate to a uniform map, and the conjugating function is precisely the inverse Rosenblatt transformation.

Notice that both the translation operator $T^{c}$ in (9) and the triangle map in (10) are uniform maps on the unit interval [0,1]. Thus, the solutions to the IFPP given in Equations (8) and (13) are examples of the general solution form in (18). In particular, while the solution to the IFPP in (13) was derived assuming the symmetry of the target density ρ(·), (13) actually gives a solution of the IFPP for *any* density ρ(·). Unimodal maps of this form were derived in [8].

Computed examples of solutions to the IFPP given by the factorization (18) are presented in Section 5 in one dimension and in Section 6 in two dimensions. High-dimensional calculations are discussed in Section 7.

### 4.3. Properties of M from U

Many properties of the map *M* are inherited from the uniform map *U*.

When *R* and $R^{-1}$ are continuous, *M* is continuous if and only if *U* is continuous. In one dimension, the monotonicity of the CDF and IDF implies that the number of modes of *U* equals the number of modes of *M*; in particular, *M* is unimodal if and only if *U* is unimodal.

Constructing iterative maps with a specific periodicity of the orbit is possible through the use of translation operators $T^{c}$ as uniform maps, defined in Equation (9). First, consider maps in one dimension on [0,1]. If the shift c≠0 and c∉Q, the map is aperiodic. However, in the case that c≠0 and c∈Q such that

(19) $$c=\frac{N}{D}$$

with N,D∈N and gcd(N,D)=1, then the map is periodic with periodicity *D*, and iterative maps constructed with $U=T^{c}$ exhibit the same periodicity. These properties may be extended to multi-dimensional settings when the translation constant *c* is a vector of shifts in each coordinate direction, as used in rank-one lattice rules for quasi-Monte Carlo integration [26].

The factorization in Theorem 1 also shows that performing an iteration $x_{n+1}=M\left(x_{n}\right)$ with an iterative map *M* on the space *X* is equivalent to applying the corresponding uniform map $z_{n+1}=U\left(z_{n}\right)$ on the space ${\left[0,1\right]}^{d}$ through the transformations *R* and $R^{-1}$, as indicated in the following (commuting) diagram.

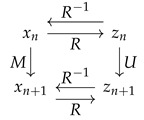

Using this commuting property, it is straightforward to prove the following lemma:

**Lemma 1.**  *For given distribution ρ, let R be a Rosenblatt transformation for ρ. Let $M=R^{-1}\circ U\circ R$ be a solution of the IFFP, as guaranteed by Theorem 1, where U is a uniform map. Then*

(20) $$O_{M}^{+}\left(x_{0}\right)=R^{-1}\left(O_{U}^{+}\left(R\left(x_{0}\right)\right)\right).$$

Thus, instead of iterating *M* on the space *X* to produce the sequence $\left\{x_{1},x_{2},x_{3},\ldots \right\}$, Equation (20) shows that one can iterate the map *U* on the space ${\left[0,1\right]}^{d}$ to produce the sequence $\left\{z_{1},z_{2},z_{3},\ldots \right\}$ and then evaluate $x_{n}=R^{-1}z_{n}$, n=1,2,… to produce exactly the same sequence on *X*. Since the map *M* is mixing or ergodic if and only if the uniform map *U* is mixing or ergodic, respectively, in this sense, the mixing and ergodicity of *M* is inherited from *U*. When the uniform map *U* satisfies the stronger condition that $\mathrm{Unif}({\left[0,1\right]}^{d})$ is the equilibrium distribution, *U* is called an *exact map* [2].

Using the expansion in Equation (20), we see that *M* is deterministic or stochastic if and only if *U* is deterministic or stochastic, respectively. Even though we do not consider stochastic maps here, we note that, for stochastic maps, iterations of *M* are correlated or independent if and only if iterations of *U* are correlated or independent, respectively.

Some other properties that are and are not preserved by the transformation from *U* to *M* are discussed in [7].

## 5. Examples in One Dimension

### 5.1. Uniform Maps on [0,1]

**Figure 3:**
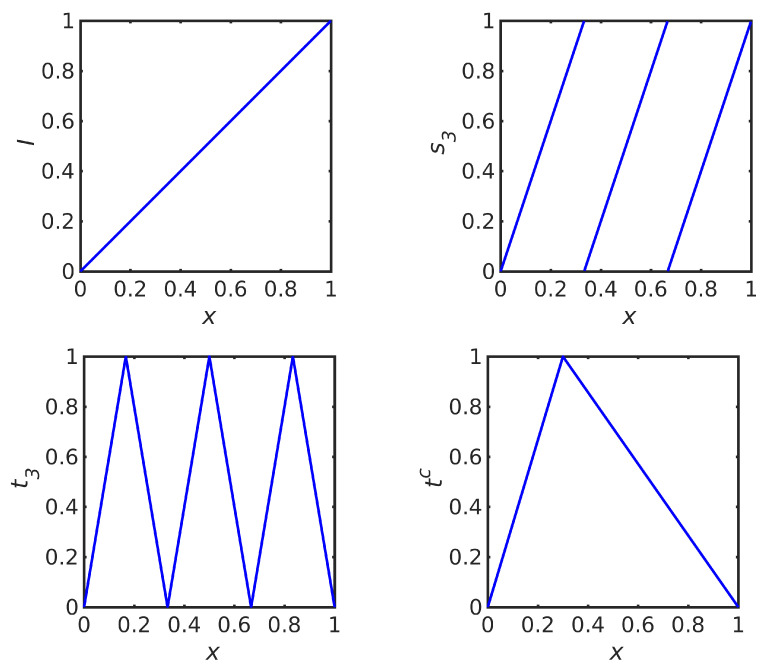
Four examples of uniform maps on [0,1]. (**Top, left**) identity map. (**Top, right**) *ℓ* periods of a sawtooth wave for ℓ=3. (**Bottom, left**) *ℓ* periods of a triangle wave for ℓ=3. (**Bottom, right**) asymmetric triangle for c=0.3.

We have already encountered three uniform maps on the interval [0,1], namely the identity map I(x)=x (Figure 3 (top, left)) and the translation operator (9) (Figure 1 (left)), that have the Lyapunov exponent h=0, and the triangle map (Figure 1 (right)) with the Lyapunov exponent h=log2.

Some further elementary uniform maps on [0,1] and associated Lyapunov exponents are listed as follows:

- *ℓ* periods of a sawtooth function on [0,1] (Figure 3, top-right, for l=3 periods)
  (21) $$s_{\ell }\left(x\right)=\ell *x-\left\lfloor \ell *x\right\rfloor ,$$
  with the Lyapunov exponent h=logℓ; (the two-period sawtooth map $s_{2}$ is also called the Bernoulli map, and its orbit $O^{+}\left(x\right)$ is the dyadic transformation)
- *ℓ* periods of a triangle function on [0,1] (Figure 3 (bottom, left) for l=3 periods)
  (22) $$t_{\ell }\left(x\right)=1-2\left|s_{\ell }\left(x\right)-1/2\right|.$$
  with the Lyapunov exponent h=log2ℓ; (this is the “broken linear transformation” in [7] of order p=2ℓ)
- The asymmetric triangle, for c∈(0,1) (Figure 3 (bottom, right) for c=0.3)
  (23) $$t^{c}\left(x\right)=\left\{\begin{array}{cc} \frac{x}{c} & 0\leq x\leq c\\ \frac{1-x}{1-c} & c\leq x\leq 1 \end{array}\right.$$
  with the Lyapunov exponent 0≤h=−clogc−(1−c)log(1−c)≤log2.

Obviously, many more uniform maps are possible. Further examples can be formed by partitioning the domain and range of any uniform map and then permuting the subintervals. Many existing “matrix-based” methods for constructing solutions to the IFPP can be viewed as examples of such a partition-and-permute of an elementary uniform map [27]. Uniform maps of other forms are developed in [28] from two-segmental Lebesgue processes, producing uniform maps that are curiously non-linear.

**Lemma 2.**  *The composition of uniform maps is also a uniform map; i.e., if $U_{1}$ and $U_{2}$ are uniform maps, then so is $U=U_{1}\circ U_{2}$.*

An example is $t_{\ell }$, which can be constructed as the composition $t_{\ell }=s_{\ell }\circ t_{1}$.

We mentioned the numerical artifacts that can occur with finite-precision arithmetic, particularly when implementing maps on a binary computer and when the endpoints of the interval *X* and constants in the maps have exact binary representations. Computation was performed in MatLab implementing IEEE Standard 754 for the double-precision binary floating-point format. We avoided these artifacts by composing the stated uniform map with the translation $T^{c}$ for $c=1/3\times 10^{-9}$ that does not have a finite binary representation. This small shift is indiscernible in the graphs of the maps.

### 5.2. Ramp Distribution

To give a concrete example of the solution in (13) for a distribution without reflexive symmetry, we consider the ramp distribution with PDF

(24) $$\rho_{\mathrm{ramp}}\left(x\right)=2x$$

that has CDF

$$F_{\mathrm{ramp}}\left(x\right)=x^{2}.$$

**Figure 4:**
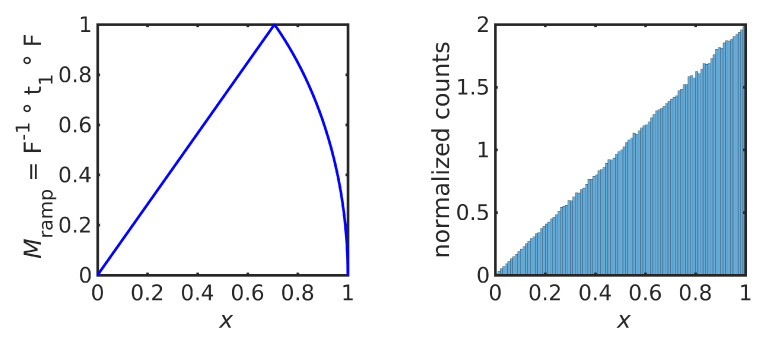
Iterative map $M_{\mathrm{ramp}}$ in (25) (**left**) and a normalized histogram of $1\times 10^{6}$ iterations that approximates the equilibrium PDF (**right**).

We produce a unimodal, continuous map by choosing the uniform map $t_{1}$, as in (13), to give

(25) $$M_{\mathrm{ramp}}=\left\{\begin{array}{cc} \sqrt{2}x & 0\leq x\leq \frac{1}{\sqrt{2}}\\ \sqrt{2}\sqrt{1-x^{2}} & \frac{1}{\sqrt{2}}\leq x\leq 1 \end{array}\right.$$

as shown in Figure 4 (left). Figure 2 (right) shows a normalized histogram of $10^{6}$ iterations of $M_{\mathrm{ramp}}$ starting at x=0.3, confirming that the orbit of $M_{\mathrm{ramp}}$ converges to the desired ramp distribution, as guaranteed by Theorem 1. The numerical implementation avoids finite-precision effects, as discussed earlier.

The estimated Lyapunov exponent for this map is h≈1.040035, which is greater than the Lyapunov exponent for the inducing triangular map $t_{1}$, which is log2≈0.693147.

**Figure 5:**
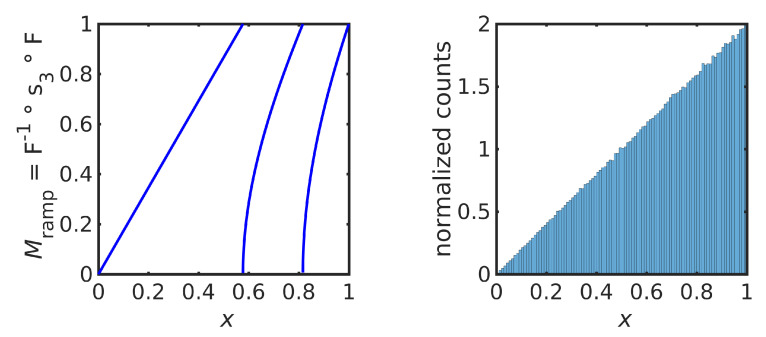
Iterative map $M_{\mathrm{ramp}}$ in (25) (**left**) and a normalized histogram of $1\times 10^{6}$ iterations that approximates the equilibrium PDF (**right**).

Using a different uniform map gives a different solution to the IFPP. For example, choosing $s_{3}$ gives the map $M=F^{-1}\circ s_{3}\circ F$, as shown in Figure 5 (left). A normalized histogram over an orbit of $10^{6}$ iterations is shown in Figure 5 (right), confirming that this map is also ergodic for $\rho_{\mathrm{ramp}}$.

The estimated Lyapunov exponent for this map is h≈1.098612, which is the same numerical value as the Lyapunov exponent for the sawtooth map $s_{3}$, which is log3≈1.098612.

### 5.3. The Logistic Map and Alternatives

The logistic map, as mentioned in the introduction, is

(26) $$M_{\log}\left(x\right)=4x\left(1-x\right).$$

The equilibrium distribution of this map

(27) $$\rho_{\log}\left(x\right)=\frac{1}{\pi \sqrt{x(1-x)}},$$

can be easily verified by substituting into the FP Equation (6). The CDF of $\rho_{\log}\left(x\right)$ is

(28) $$F_{\log}\left(x\right)=\int_{0}^{x}\rho_{\log}\left(x^{\prime }\right)dx^{\prime }=\frac{2}{\pi }\arcsin\left(\sqrt{x}\right),$$

and the IDF is

(29) $$F_{\log}^{-1}\left(x\right)=\sin^{2}\left(\frac{\pi x}{2}\right)=\frac{1}{2}\left(1-\cos\left(\pi x\right)\right).$$

**Figure 6:**
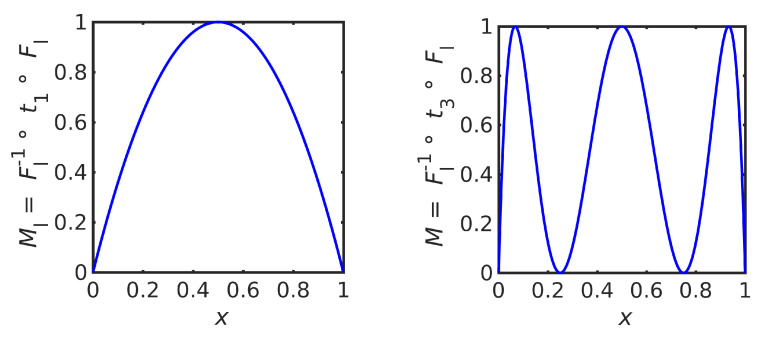
Logistic map $M_{\log}$, that equals $M_{1}=F_{\log}^{-1}\circ t_{1}\circ F_{\log}$ (**left**), and the map $M_{3}=F_{\log}^{-1}\circ t_{3}\circ F_{\log}$ that has the same equilibrium distribution (**right**).

The logistic map (26) is induced by the factorization (18) by choosing the triangle map $t_{1}$ as a uniform map; i.e., substituting the CDF (28) and IDF (29) into (13) (see Figure 6 (left)). Equivalently, one may note that the logistic map (26) is transformed into the triangle map $t_{1}$ by the change of variables $z=F^{-1}\left(x\right)$; in the language of [7], $M_{\log}$ and $t_{1}$ are *conjugate* dynamical laws.

Other iterative maps that preserve the same equilibrium distribution (27) can be constructed by choosing another uniform map, such as *ℓ* periods of a triangle function (22). This gives the iterative maps

(30) $$M_{\ell }=F_{\log}^{-1}\circ t_{\ell }\circ F_{\log}=\sin^{2}\left(2\ell \arcsin\left(\sqrt{x}\right)\right),\ell \geq 1,$$

that coincide with the *n*th power of the logistic map (26) for $\ell =2^{n-1}$. Figure 6 (right) shows the map which preserves the same equilibrium distribution as the logistic map but induced by the uniform map $t_{3}$. Since 3 is not of the form $2^{n-1}$, this map is not simply a power of the logistic map.

The theoretical value of the Lyapunov exponent of the map in (30) is log2ℓ, using (5). Table 1 gives the theoretical values of the Lyapunov exponent and experimentally calculated values using 10,000 iterations, as in (4), for some values of *ℓ*.

## 6. Two Examples in Two Dimensions

### 6.1. Uniform Maps on ${\left[0,1\right]}^{2}$

Two well-known examples of uniform maps in the two-dimensional unit square, $X={\left[0,1\right]}^{2}$, are the baker’s map

(31) $$U_{b}\left(x_{1},x_{2}\right)=\left(2x_{1}\;\mathrm{mod}\;1,\frac{x_{2}+u\left(x_{1}-\frac{1}{2}\right)}{2}\right),$$

where *u* is the unit step function, and the Arnold cat map

(32) $$U_{A}\left(x_{1},x_{2}\right)=\left(\left(2x_{1}+x_{2}\right)\;\mathrm{mod}\;1,\left(x_{1}+x_{2}\right)\;\mathrm{mod}\;1\right).$$

Other uniform maps in d>1 dimensions may be formed by 1 dimensional uniform maps acting on each coordinate, giving the coordinate-wise uniform map

(33) $$U\left(x\right)=\left(U_{1}\left(x_{1}\right),U_{2}\left(x_{2}\right),\ldots ,U_{n}\left(x_{d}\right)\right)$$

where $U_{i}\left(x\right)$, i=1,2,…,d, are uniform maps in 1 dimension. We use the baker’s map (31) and a coordinate-wise uniform map in the 2 dimensional examples that follow.

### 6.2. Checker-Board Distribution

**Figure 7:**
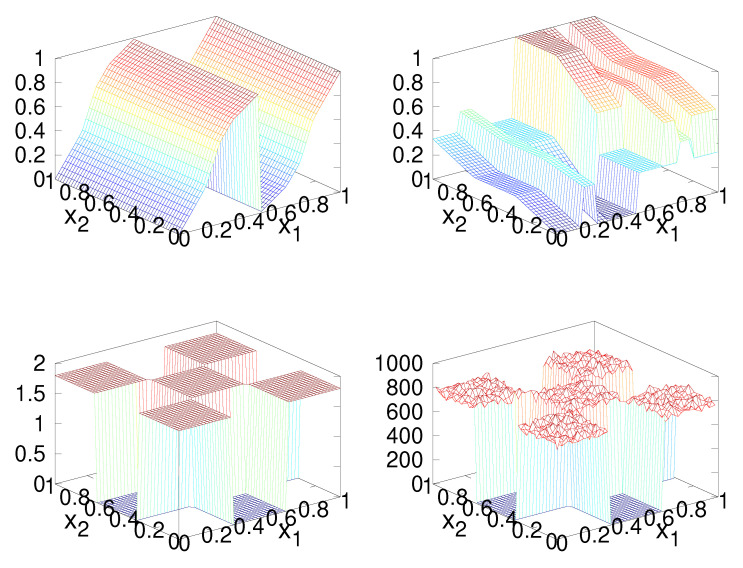
Iterated function constructed with *U* being the baker’s transformation, a histogram of iterations, and the checker board distribution. The $x_{1}$ part of the constructed map (**top-left**), the $x_{2}$ part of the constructed map (**top-right**), the checker board distribution (**bottom-left**), and a histogram of iterates of the constructed map (**bottom-right**) are shown.

This example demonstrates the construction of a map in two-dimensions that targets a checker board distribution, as shown in Figure 7 (bottom-left), using the factorization (18).

**Figure 8:**
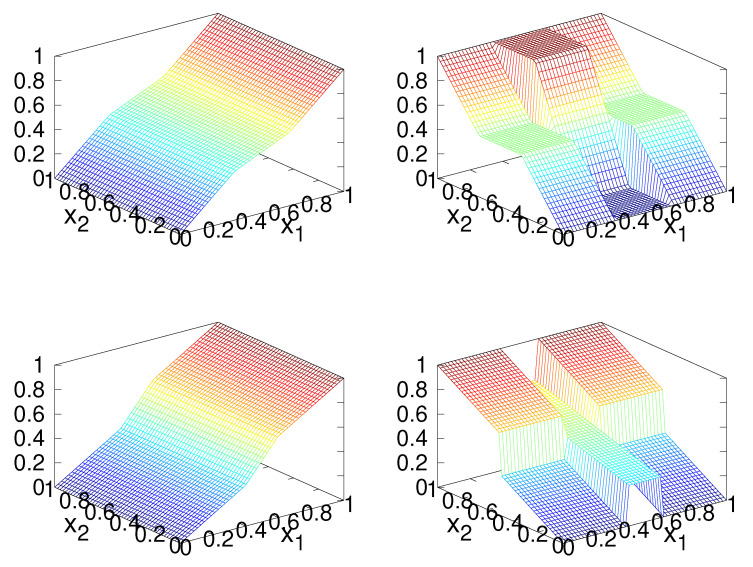
Plots of the Rosenblatt transformation *R* and its inverse $R^{-1}$ for the checker-board distribution. The top row shows the components of *R*: first component (**left**) and second component (**right**). The bottom row shows the components of $R^{-1}$: first component (**left**) and second component (**right**).

The first step in constructing a solution to the IFPP for this distribution is to construct the forward and inverse Rosenblatt transformations, which requires the marginal density functions (17), which may be evaluated analytically in this case. A plot of the two components of the functions *R* and $R^{-1}$ is shown in Figure 8.

We construct two solutions to the IFPP, each induced by choosing a particular uniform map: the first is the baker’s map (31), and the second is a component-wise uniform map (33) with an asymmetric triangle map (23) acting on each component,

(34) $$U\left(x_{1},x_{2}\right)=\left(U_{1}\left(x_{1}\right),U_{2}\left(x_{2}\right)\right)$$

where $U_{1}=t^{c}$ for c=0.3 and $U_{2}=t^{c}$ with c=0.9.

Figure 7 (top row) shows the two components of the map induced by the baker’s map, the checker-board distribution (bottom-left), and a histogram of $10^{6}$ iterations (bottom-right) showing that the map does indeed converge in distribution to the desired distribution.

**Figure 9:**
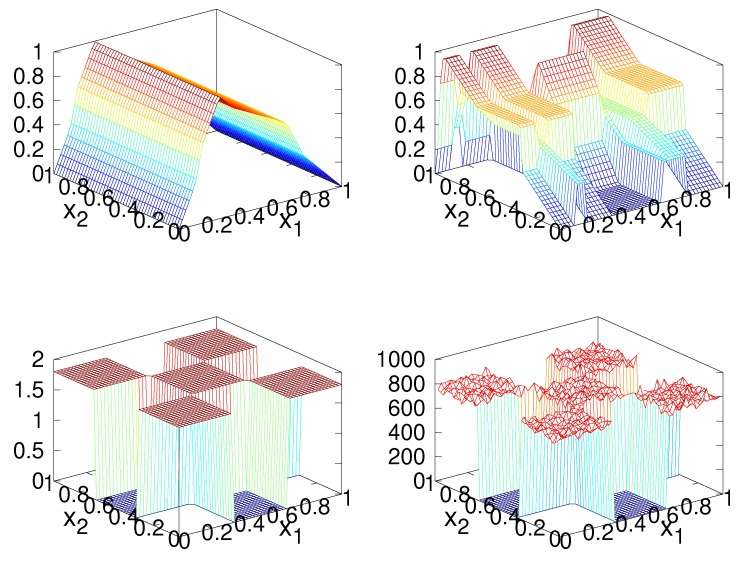
Same as Figure 7, except *M* is constructed with *U* being component-wise asymmetric triangular maps.

Figure 9 (top row) shows the two components of the map constructed using the two component-wise asymmetric triangular maps, the checker-board distribution (bottom-left), and a histogram of $10^{6}$ iterations (bottom-right) showing that the map also converges in distribution to the desired distribution, and hence is also a solution to the IFPP.

### 6.3. A Numerical Construction

**Figure 10:**
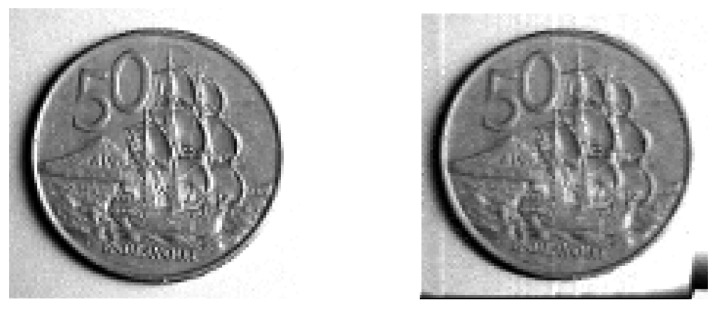
A normalized greyscale image of a coin used as the target distribution, and a normalized histogram of iterations of the map targeting this distribution. Original image (**left**), and normalized histogram of $10^{6}$ iterations (**right**).

The numerical implementation of the factorized solution (18) is not difficult in a small number of dimensions. In this section, we present an example of numerical implementation using a normalized greyscale image of a pre-2006 New Zealand 50 cent coin, piecewise constant over pixels, as the target distribution; see Figure 10 (left). The marginal distributions (17) are evaluated as a linear interpolation of cumulative sums over pixel values, and thus the CDF and then forward and inverse Rosenblatt transformations follow as in Section 4.1. The uniform map was produced as component-wise univariate translation maps, specifically

$$U\left(x_{1},x_{2}\right)=\left(U_{1}\left(x_{1}\right),U_{2}\left(x_{2}\right)\right)$$

where $U_{1}=T^{c}$ for c=0.6 and $U_{2}=T^{c}$ with c=0.2. The resulting map is given by Equation (18).

Figure 10 (right) shows a normalized histogram, binned to pixels, of $10^{6}$ iterations of this map. As can be seen, the estimated PDF from the orbit of this map does reproduce the image of the coin. However, there are also obvious artifacts near the edge of the image showing that the mixing could be better. We conjecture that a chaotic uniform map would produce better mixing and fewer numerical artifacts.

## 7. Summary and Discussion

We have shown that the solution of the IFPP—finding an iterative map with a given invariant distribution—can be constructed from uniform maps through the factorization established in Theorem 1,

$$M=R^{-1}\circ U\circ R$$

where *R* denotes the Rosenblatt transformation that has a Jacobian determinant equal to the density function of the invariant distribution. In one dimension, *R* is exactly the CDF of the given distribution, meaning that the factorization generalizes existing one-dimensional solutions to the setting of arbitrary multi-variate distributions. The factorization also shows the relationship between arbitrary iterative maps and uniform maps; i.e., given a Rosenblatt transformation, the solution of the IFPP is equivalent to the choice of a uniform map that has $\mathrm{Unif}({\left[0,1\right]}^{d})$ as an invariant distribution.

We find the factorization (18) appealing as it shows that the solution of the IFPP for arbitrary distributions, and in multiple dimensions, is reduced to two standard and well-studied problems; i.e., constructing the Rosenblatt transformation (or CDF in one dimension) and designing a uniform map. It is therefore surprising to us that the factorization (18), and more generally the Rosenblatt transformation, appears not to be widely used in the study of chaotic iterated functions and the IFPP. Grossmann and Thomae [7], in one of the earliest studies of the IFPP, essentially derived the factorization (18) by introducing conjugate maps and establishing the relation (in their notation) that $\rho^{*}\left(x\right)=dh^{-1}\left(x\right)/dx$, where $\rho^{*}$ is the invariant distribution and *h* is the conjugating function; see [7] (Figure 3). It is a small step to identify that *h* is the IDF, generalized in multiple dimensions by the inverse Rosenblatt transformation. However, the connection was not made in [7], despite the Rosenblatt transformation having been already known in statistics for some decades [18].

We constructed solutions to the IFPP for distributions with a special reflexive symmetry structure, and then with no special structure, by constructing the Rosenblatt transformation and its inverse for some examples in one and two dimensions. For simple distributions with an analytic form, the Rosenblatt transformation may be constructed analytically, while numerically-defined distributions require the calculation of the marginal distributions (17) using numerical techniques.

Although this factorization and construction is applicable to high-dimensional problems, the main difficulty is obtaining all necessary marginal densities, which requires the high-dimensional integral over $x_{k+1}\ldots x_{d}$ in (17). In general, this calculation can be extremely costly. Even a simple discretization of the PDF ρ, or of the argument of the marginal densities (17), leads to a cost that grows exponentially with the number of dimensions.

To overcome this cost, Dolgov et al. [25] precomputed an approximation of $\rho (x_{1},\ldots ,x_{d})$ in a compressed tensor train representation that allows the fast computation of integrals in (17) and subsequent simulation of the inverse Rosenblatt transformation $R^{-1}$ from the conditionals in (17) and showed that computational cost scales *linearly* with dimension *d*. Practical examples presented in [25], in dimension d≤32, demonstrate that operation by the forward and inverse Rosenblatt transformations is computationally feasible for multivariate problems with no special structure.

Finding a solution of the IFPP with desired properties is reduced to a standard problem of designing a uniform map on ${\left[0,1\right]}^{d}$, for which there are many existing efficient options. For example, standard computational uniform random number generators, which produce pseudo-random sequences of numbers, are one such existing uniform map, as are the quasi-Monte Carlo rules mentioned earlier [26]. These induce pseudo-random and quasi-Monte Carlo sequences, respectively, on the space *X* via the inverse Rosenblatt transformation $R^{-1}$ [29]. Both these schemes were demonstrated in practical high-dimensional settings in [25].

The RHS of Equation (20) in d=1 dimension is exactly the standard computational route for implementing inverse cumulative transformation sampling from ρ, since computational uniform pseudo-random number generators perform a deterministic iteration on [0,1] to implement a uniform map [29]. For d>1, the RHS of Equation (20) is the conditional distribution method that generalizes the inverse cumulative transformation method, as mentioned above [22,23,24,25]. Thus, Lemma 1 shows that the standard computational implementation of both the inverse cumulative transformation method in d=1 and the conditional distribution method in d>1 is equivalent to implementing a solution to the IFFP. In this sense, computational inverse cumulative transformation sampling from ρ can be viewed as the prototype for all iterative maps that target the distribution ρ, with each ergodic sequence corresponding to a particular choice of uniform map.

We mentioned that the Rosenblatt transformation associated with a given distribution ρ is not unique. Actually, any two Rosenblatt transformations for ρ are related by a uniform map, as shown in the following Lemma.

**Lemma 3.**  *If $R_{1}$ is a Rosenblatt transformation for ρ then $R_{2}$ is a Rosenblatt transformation for ρ if and only if*

$$R_{2}=U\circ R_{1}$$

*for some uniform map U.*

**Proof.**  (⇒) Since $R_{1}$ and $R_{2}$ are Rosenblatt transformations for ρ, then $U=R_{2}\circ R_{1}^{-1}$ is a uniform map and $R_{2}=U\circ R_{1}$. (⇐) If $R_{2}=U\circ R_{1}$ then if x∼ρ, $R_{1}\left(x\right)∼\mathrm{Unif}\left({\left[0,1\right]}^{d}\right)$ and $U\circ R_{1}\left(x\right)∼\mathrm{Unif}\left({\left[0,1\right]}^{d}\right)$, and so $R_{2}$ is a Rosenblatt transformation. □

Thus, any Rosenblatt transformation *R* may be written as $R=U\circ R_{0}$ for some uniform map *U* and a fixed Rosenblatt transformation $R_{0}$.

The Rosenblatt transformations that map any distribution to the uniform distribution on the hypercube may also be used to understand mappings between spaces that are designed to transform one distribution to another, such as the “transport maps” developed in [30]. Consider distributions $\rho_{A}$ and $\rho_{B}$, with Rosenblatt transformations $R_{A}$ and $R_{B}$, respectively, that may be related as in the following diagram:

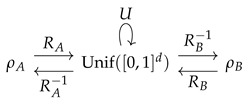

The diagram provides a proof of the following lemma, which generalizes the factorization shown in Theorem 1.

**Lemma 4.**  *A map M satisfies $x∼\rho_{A}\Rightarrow M\left(x\right)∼\rho_{B}$ if and only if it can be written as $M=R_{B}^{-1}\circ U\circ R_{A}$, where $R_{A}$ and $R_{B}$ are Rosenblatt transformations for $\rho_{A}$ and $\rho_{B}$, respectively, and U is a uniform map.*

Thus, for given Rosenblatt transformations, the choice of a map that maps samples from $\rho_{A}$ to samples from $\rho_{B}$ is equivalent to the choice of a uniform map. Alternatively, if a fixed uniform map is selected, such as the identity map, the choice of map *M* is completely equivalent to the choice of Rosenblatt transformations. This factorization also shows that the equivalence class of conjugate maps, noted in [7] for each dimension *d*, is generated by the uniform maps, and each member of the equivalence class contains maps that target each distribution, when the associated Rosenblatt transformation satisfies the mild conditions to be a conjugating function as defined in [7].

## Figures and Tables

**Table 1:** Experimental $h_{e}$ and theoretical $h_{t}$ Lyapunov exponents for the maps in Figure 6 for a range of *ℓ*, given to six decimal places.

*ℓ*	$h_{e}$	$h_{t}$
1	0.692819	0.693147
2	1.386284	1.386294
4	2.079430	2.079442
$2^{24}$	17.328594	17.328680
$2^{39}$	27.725713	27.725887

## Abbreviations

The following abbreviations are used in this manuscript:

PDF	Probability density function
FP	Frobenius–Perron
IFPP	Inverse Frobenius–Perron problem
CDF	Cumulative distribution function
IDF	Inverse distribution function

## References

[1] Dorfman J. (1999). Cambridge Lecture Notes in Physics: An Introduction to Chaos in Nonequilibrium Statistical Mechanics.

[2] Lasota A., Mackey M.C. (1994). Chaos, Fractals, and Noise.

[3] Ulam S., von Neumann J. (1947). On Combination of Stochastic and Deterministic Processes. Bull. Am. Math. Soc..

[4] May R. (1976). Simple mathematical models with very complicated dynamics. Nature.

[5] Liu J.S. (2001). Monte Carlo Strategies in Scientific Computing.

[6] Klus S., Koltai P., Schütte C. (2016). On the numerical approximation of the Perron–Frobenius and Koopman operator. J. Comput. Dyn..

[7] Grossmann S., Thomae S. (1977). Invariant distributions and stationary correlation functions of one-dimensional discrete processes. Zeitschrift für Naturforschung A.

[8] Ershov S.V., Malinetskii G.G. (1988). The solution of the inverse problem for the Perron–Frobenius equation. USSR Comput. Math. Math. Phys..

[9] Diakonos F., Schmelcher P. (1996). On the construction of one-dimensional iterative maps from the invariant density: The dynamical route to the beta distribution. Phys. Lett. A.

[10] Diakonos F., Pingel D., Schmelcher P. (1999). A stochastic approach to the construction of one-dimensional chaotic maps with prescribed statistical properties. Phys. Lett. A.

[11] Pingel D., Schmelcher P., Diakonos F. (1999). Theory and examples of the inverse Frobenius–Perron problem for complete chaotic maps. Chaos.

[12] Bollt E.M. (2000). Controlling chaos and the inverse Frobenius–Perron problem: Global stabilization of arbitrary invariant measures. Int. J. Bifurc. Chaos.

[13] Nie X., Coca D. A new approach to solving the inverse Frobenius–Perron problem. Proceedings of the 2013 European Control Conference (ECC).

[14] Nie X., Coca D. (2018). A matrix-based approach to solving the inverse Frobenius–Perron problem using sequences of density functions of stochastically perturbed dynamical systems. Commun. Nonlinear Sci. Numer. Simul..

[15] Rogers A., Shorten R., Heffernan D., Naughton D. (2008). Synthesis of Piecewise-Linear Chaotic Maps: Invariant Densities, Autocorrelations, and Switching. Int. J. Bifurc. Chaos.

[16] Wei N. (2015). Solutions of the Inverse Frobenius–Perron Problem. Master’s Thesis.

[17] Ulam S.M. (1960). A Collection of Mathematical Problems.

[18] Rosenblatt M. (1952). Remarks on a Multivariate Transformation. Ann. Math. Stat..

[19] Gaspard P. (1998). Chaos, Scattering and Statistical Mechanics.

[20] Varberg D.E. (1971). Change of variables in multiple integrals. Am. Math. Mon..

[21] Györgyi G., Szépfalusy P. (1984). Fully developed chaotic 1-d maps. Z. für Physik B Condens. Matter.

[22] Johnson M. (1987). Multivariate Statistical Simulation.

[23] Devroye L. (1986). Non-Uniform Random Variate Generation.

[24] Hörmann W., Leydold J., Derflinger G. (2004). Automatic Nonuniform Random Variate Generation.

[25] Dolgov S., Anaya-Izquierdo K., Fox C., Scheichl R. (2020). Approximation and sampling of multivariate probability distributions in the tensor train decomposition. Stat. Comput..

[26] Dick J., Kuo F.Y., Sloan I.H. (2013). High-dimensional integration: The quasi-Monte Carlo way. Acta Numer..

[27] Rogers A., Shorten R., Heffernan D.M. (2004). Synthesizing chaotic maps with prescribed invariant densities. Phys. Lett. A.

[28] Huang W. (2005). Characterizing chaotic processes that generate uniform invariant density. Chaos Solitons Fractals.

[29] Gentle J.E. (2003). Random Number Generation and Monte Carlo Methods.

[30] Parno M.D., Marzouk Y.M. (2018). Transport map accelerated Markov chain Monte Carlo. SIAM/ASA J. Uncertain. Quantif..

